# Dilatation Therapy and Demographic Characteristics Significantly Influence the Amount of Propofol for Therapeutic Endoscopic Retrograde Cholangiography

**DOI:** 10.1155/2019/4793096

**Published:** 2019-07-01

**Authors:** Christoph A. Schmidt, Carsten Keil, Martha M. Kirstein, Frank Lehner, Michael P. Manns, Thomas von Hahn, Tim O. Lankisch, Torsten Voigtländer

**Affiliations:** ^1^Department of Gastroenterology, Hepatology and Endocrinology, Hannover Medical School, Hannover, Germany; ^2^Department of General, Visceral and Transplantation Surgery, Hannover Medical School, Hannover, Germany

## Abstract

**Background and Study Aims:**

Patients undergoing therapeutic endoscopic retrograde cholangiography (ERC) may require different amounts of sedative agents depending on demographic characteristics, indication of ERC, and/or endoscopic intervention.

**Patients and Methods:**

We retrospectively analyzed all patients undergoing therapeutic ERC from 2008 – 2014 who received deep sedation with propofol ± midazolam.

**Results:**

A total of 2448 ERC procedures were performed in 781 patients. The cumulative per procedure propofol dose in the different groups was as follows: PSC 479 mg (±256), bile duct stones 356 mg (±187), benign stenosis/cholestasis 395 mg (±228), malignant stenosis 401 mg (±283), and postliver transplant complications 391 mg (±223) (p < 0.05). Multivariable analysis showed that dilatation therapy (p = 0.001), age (p = 0.001), duration of the intervention (p = 0.001), BMI (p = 0.001), gender (p = 0.001), platelet count (p = 0.003), and bilirubin (p = 0.043) influence independently the propofol consumption.

**Conclusions:**

Demographic characteristics and endoscopic interventions have a distinct influence on the amount of sedation required for therapeutic ERC. Although the sedation-associated complication rate is low optimization of sedative regimens is a prime goal to further reduce adverse events of therapeutic ERC.

## 1. Introduction

Endoscopic retrograde cholangiography (ERC) is an invasive procedure which may be associated with patient discomfort, abdominal pain, and potentially life-threatening complications [1, 2]. Consequently, sedation is an essential aspect of ERC as it may reduce stress, anxiety, and pain of patients leading to higher acceptability and improved conditions for the procedure [3, 4]. Main indications for therapeutic ERC are management of benign and malignant bile duct strictures, pancreatic diseases, removal of bile duct stones, treatment of biliary complications after liver transplantation and others [5–8]. One established sedation regimen for ERC includes the application of propofol ± midazolam [9].

Major factors influencing the pharmacokinetic profile and clinical effects of propofol are gender, weight, and age [3]. These factors and changes in the need of sedation for different endoscopic procedures are well described [10–12]. Increasing evidence suggest that distinct patient groups may require different amounts of sedative drugs for completion of therapeutic ERC independent of gender, weight, and age. In a prior study we could show that patients with primary sclerosing cholangitis (PSC) require more sedative agents for ERC compared to control groups [13]. These findings prompted us to further analyze the efficacy, safety, and factors influencing the required doses of anesthetic drugs in patients undergoing ERC at our institution.

## 2. Patients/Material and Methods

All patients presenting for ERC to the endoscopic unit of Hannover Medical School between 2008 and 2014 were retrospectively analyzed. Patients who underwent an ERC procedure in general anesthesia were excluded from the study as well as patients receiving opioids during the intervention. In case of repetitive endoscopic examinations, all presentations were included in the analysis. Demographic characteristics, duration (minutes (min)) and time point of the intervention, underlying diseases, and the application rate of the anesthetics (amount of anesthetics) were extracted from the endoscopy database. Deep sedation was performed by intermittent bolus application of propofol with or without midazolam as premedication. No additional application of analgesics was performed. The sedation was administered and monitored by the endoscopists (regularly one endoscopist for examination and one endoscopist for sedation). Routinely, drugs were given intravenously. For induction, approximately 0,05 mg of midazolam per kg/body weight and 0,5 mg of propofol per kg/ body weight were given. For maintenance, regular incremental boluses were applicated. An infusion pump was not regularly used for propofol sedation. During ERC a deep sedation level (Ramsay sedation scale of 5-6) with maintained cardiovascular and respiratory function was targeted and controlled by a gastroenterologist who is expert and trained in advanced life support skills.

All physicians performing ERC at our institution are experienced endoscopists (> 300 examinations, > three years of regular ERC performance). The study was approved by the local institutional Ethics Committee (Ethics Committee of Hannover Medical School). Data were analyzed retrospectively and anonymously. Consequently, no written informed consent is available or necessary. The study protocol conforms to the ethical guidelines of the 1975 Declaration of Helsinki, as reflected in a prior approval (June 2017) by the institution's human research committee.

## 3. Statistical Analysis

Baseline characteristics at the time of the first intervention are presented as absolute and relative frequencies for categorical variables and mean ± standard deviation, unless denoted otherwise. Our study analyzes the influence of different variables (endoscopic, demographic) on the propofol dose for completion of therapeutic ERC. Propofol dose served as the dependent variable. A logarithmic transformation was applied to all metric variables which showed a skewed distribution on the original scale. In case of more than 2 values for categorical variables a dummy transformation was performed.

In a first step we checked the influence of each independent variable separately in univariate models. Subsequently, significant (p < 0.05) variables were entered into a multivariable linear regression model and a backward selection based on the Wald statistic was performed. All analyses are of explorative character and no multiplicity correction was applied. The software used was the SPSS Statistical Package (version 19.0, SPSS Inc., Chicago, IL).

## 4. Results

A total of 2448 ERC procedures were performed in 781 patients during the study period. Indication for ERC was PSC in 18.7% (458/2448), bile duct stones in 7% (171/2448), benign stenoses/cholestasis in 20% (490/2448), malignant stenoses in 17.1% (419/2448), and postliver transplant complications in 37.2% (910/2448) of the interventions. A median of 2 (1-4) examinations were performed per patient. The diagnoses of the study population were as follows: PSC 19.6% (153/781), bile duct stones 11.4% (89/781), benign stenoses/cholestasis 24.8% (194/781), malignant stenoses 21.6% (169/781), and postliver transplant complications in 22.4% (175/781).

The mean age in the PSC cohort was 42 years (±12), 64 years (±15) for patients with bile duct stones, 56 years (±15) for patients with benign stenoses/cholestasis, 65 years (±11) for patients with malignant stenoses, and 51 years (±12) for patients with postliver transplant complications (p <0.0001), respectively. The mean body mass index (BMI) was 24.4 kg/m^2^ (±3.8), 26.2 kg/m^2^ (±4.6), 24.8 kg/m^2^ (±5.3), 24.4 kg/m^2^ (±3.8), 24.4 kg/m^2^ (±4.8), and 25.3 kg/m^2^ (±5) for the aforementioned groups (p = 0.258). Demographic characteristics of the study cohort are illustrated in Table 1.

A papillotomy was performed or had already been performed prior to the study period in 71% of the patients with PSC, 81% of patients with bile duct stones, 70% of patients with benign stenoses/cholestasis, 60% of patients with malignant stenoses, and 84% of patients with postliver transplant complications (p < 0,001). A dilatation therapy was applied in 30%, 3%, 13%, 27%, and 42% for the aforementioned groups, respectively. All endoscopic interventions and measures for the different patient groups are given in Table 2.

The mean duration of the endoscopic interventions was 49.9 min (±25.4) for all examinations. For patients with PSC the mean duration of ERC was 50 min (±24.7), for patients with bile duct stones 49 min (±26.3), for patients with benign stenoses/cholestasis 48.8 min (±25.3), for malignant stenoses 55.4 min (±27.8), and for patients with postliver transplant complications 48 min (±24) (p < 0.001). In post hoc analysis only the comparison of patients with malignant stenoses versus all other indications showed a significant difference for duration of the intervention (p < 0.05). Analysis of the duration of ERC procedures showed no progression over time by visit number for all patients (p > 0.05). Immediate endoscopic adverse events and sedation-associated complications are depicted in Table 2.

The total propofol consumption in the different groups was as follows: PSC 479 mg (±256), bile duct stones 356 mg (±187), benign stenoses/cholestasis 395 mg (±228), malignant stenoses 401 mg (±283), and postliver transplant complications 391 mg (±223) (p < 0.05). Post hoc analysis showed that patients with PSC had a significantly higher demand of propofol compared to all groups (p < 0.05). Patients with malignant stenoses received significantly more propofol than patients who were treated because of biliary stones (p = 0.033). The propofol consumption was equal over time and revealed no progression in case of repetitive ERC procedures for all patients (up to 10 interventions). Patients with PSC and postliver transplant complications received significantly more midazolam compared to all other groups (p < 0.01; PSC 5 mg (±1), postliver transplant complications 5 mg (±1); other groups 4 mg (±1)).

In order to identify independent variables which influence the propofol consumption during ERC a multivariable analysis was performed. Multivariable analysis showed that dilatation therapy (unstandardized coefficient B 29.8; 95%-confidence interval (CI) 12.4; 47.1 (p = 0.001)), age (B -4.1; 95%-CI -4.6; -3.5 (p = 0.001)), duration of the intervention (B 6.5; 95% CI 6.2; 6.8 (p = 0.001)), BMI (B 4.3; 95%-CI 2.5; 6.0 (p = 0.001)), gender (B 41.8; 95%-CI 25.4; 58.2 (p = 0.001)), platelet count (B 0.09; 95%-CI 0.03; 0.15 (p = 0.003)), and bilirubin (B -0.03; 95%-CI -0.06; -0.001 (p = 0.043)) influence independently the propofol consumption (Table 3). When the regression analysis was restricted to the first presentation of the patients (n = 781; exclusion of repeated measurements) dilatation therapy (p = 0.013), age (p = 0.001), duration of the intervention (p = 0.001), BMI (p = 0.05), gender (p = 0.008), platelet count (p = 0.017), and bilirubin (p = 0.036) were significantly associated with the propofol consumption. Patients undergoing dilatation therapy did not receive more midazolam compared to patients without dilatation (midazolam 4 mg (±1) for both groups; p > 0.05).

## 5. Discussion

Sedation for ERC is of paramount importance to facilitate complex endoscopic interventions [9]. However, sedation may be associated with severe adverse events and therefore an adequate monitoring and intervention-tailored sedation regimen is necessary [9, 13].

To our best knowledge we systematically analyzed for the first time the influence of demographic and endoscopic measures on the required amount of sedation for completion of therapeutic ERC. Multivariable analysis showed that dilatation therapy is independently associated with an increased need for sedation. In our model dilatation therapy led to an additional application of approximately 30 mg of propofol. This finding is plausible as resolving of malignant or scarred benign strictures is generally linked to patient discomfort leading to application of higher doses of sedation/analgetics. Moreover we could show that depending on the indication for ERC (PSC, benign stenoses, malignant stenoses, removal of bile duct stones, and postliver transplant complications) a dilatation therapy is more or less likely which facilitates an adaptation of the sedative regimen. Other endoscopic interventions or measures such as papillotomy, stent placement, stent size, contrast injection in the pancreatic duct, and pancreas stent placement were not independently associated with the amount of sedation in our study. On one hand this may reflect the improvement of endoscopic interventions and techniques in high-volume ERC centers which lead to shorter intervention time and reduced need for sedation. On the other hand demographic characteristics have a stronger influence on the need for sedation compared to short endoscopic interventions which is reflected by the inclusion of demographic parameters in the multivariable analysis. Naturally, an ERC with papillotomy and stent placement will lead to a longer intervention time and therefore to higher doses of sedation compared to an ERC without intervention. However, demographic parameters predominate the required amount of sedation. As shown by other studies increase in age and BMI and male gender affect the amount of sedation for endoscopic interventions [9, 11–13]. For example, male patients required 42 mg more propofol than female patients in our model. Another important factor is the intervention time. Extension of the intervention time by one minute led to an increase in the propofol consumption of 6.5 mg. These findings are of interest and importance for an adequate dosage and adaption of the sedative regimen.

Interestingly, we could show that laboratory parameters (bilirubin and platelet count) may serve as surrogate parameters which indicate the amount of the required sedation. Normal or mildly elevated bilirubin levels associated with an undilated common bile duct are known risk factors for post-ERC pancreatitis as the examination and cannulation may be more challenging [14, 15]. Comparably, we could show that higher bilirubin levels lead to lower application of sedative agents in our model. In contrast higher platelet count leads to higher doses of sedation in our model. In our centre mainly patients with advanced liver disease are treated and a low platelet count is a general observation. Lower platelet count may serve as surrogate parameter of disease severity which could lead to lower amounts of sedation in these patients due to the reduced hepatic metabolism. However, these assumptions are clearly beyond the scope of our study and have to be addressed in future studies.

The adverse event rate (cardiorespiratory events and immediate endoscopic adverse events) in our study was low and comparable to other studies [10, 16]. Nevertheless, optimization of sedative strategies to further decrease the sedation-associated adverse events for ERC remains a prime goal.

Our study is of explorative character and has some limitations. First of all the retrospective character of the study is a limitation. Our study did not analyze the postinterventional recovery time as it was not routinely documented. Moreover, the study design does not allow excluding potential confounders like concomitant use of nonsteroidal anti-inflammatory drugs, selective serotonin reuptake inhibitors, or alcohol consumption of the patients which may have an impact on the required dose of sedation for ERC. However, we thoroughly analyzed our endoscopic and patient data base to reduce potential bias. We included repetitive examinations in our analyses but also analyzed the data with restriction to the first presentation at our endoscopic unit. The results were equal in both cases.

In summary, we could show that demographic characteristics and endoscopic interventions have a distinct influence on the amount of sedation required for therapeutic ERC. Although the sedation-associated adverse event rate is low optimization of sedative regimens is a prime goal to further reduce adverse events of therapeutic ERC.

## Figures and Tables

**Table 1:** *Demographic, baseline characteristics, and laboratory parameters of the patient cohort*. Patients with different indication for endoscopic retrograde cholangiography (ERC) are compared regarding age, gender, body mass index (BMI), and laboratory parameters at day of first presentation.

		*Primary sclerosing cholangitis (n = 153)*		*Bile duct stones (n = 89)*		*Benign stenoses / cholestasis (n = 194)*		*Tumour (n = 169)*		*post-liver transplant cholangiopathy (n = 175)*		*p-value*
		value/n	SD	value/n	SD	value/n	SD	value/n	SD	value/n	SD	−
Age (years)		42	± 12	64	± 15	56	± 15	65	± 11	51	± 12	< 0.01
Gender	Female	43	−	54	−	62	−	70	−	71	−	< 0.01
	Male	110	−	35	−	132	−	99	−	104	−	
Body mass index (kg/m^2^)		24,4	± 3,8	26,2	± 4,6	24,8	± 5,3	24,4	± 4,8	25,3	± 5	0.258
C-reactive protein (mg/L)		14	± 22	39	± 52	44	± 56	49	± 56	24	± 33	< 0.01
White blood cells (/*µ*l)		7419	± 2941	7674	± 3310	9030	± 4795	8780	± 5916	6493	± 2931	< 0.01
Platelets (x10^3^/*µ*l)		291	± 141	245	± 93	274	± 131	267	± 135	220	± 146	< 0.01
Bilirubin (*µ*mol/L)		47	± 80	39	± 53	77	± 117	159	± 181	121	± 483	< 0.01
Gamma-Glutamyl-transferase (U/L)		283	± 290	441	± 496	512	± 666	799	± 819	549	± 529	< 0.01
Alanine-Aminotransferase (U/L)		107	± 144	128	± 200	89	± 134	133	± 136	124	± 151	< 0.01
Aspartate-Aminotransferase (U/L)		86	± 107	99	± 142	76	± 97	125	± 114	85	± 90	0.058

**Table 2:** *Endoscopic interventions in the patient cohort*. Patients with primary sclerosing cholangitis (n = 458), bile duct stones (n = 171), benign stenoses/cholestasis (n = 490), malignant stenoses (n = 419), and post-liver transplant complications (n = 910) are compared regarding distinct endoscopic interventions.

		*Endoscopic measures*					
		*Primary sclerosing cholangitis (n = 458)*	*Bile duct stones (n = 171)*	*Benign stenoses / cholestasis (n = 490)*	*Tumour (n = 419)*	*post-liver transplant cholangiopathy (n = 910)*	*p-value*
		n	n	n	n	n	
Papillotomy	none	131 (29%)	33 (19%)	148 (30%)	168 (40%)	147 (16%)	< 0,001
	conventional	35 (8%)	51 (30%)	70 (14%)	73 (17%)	103 (11%)	
	needle-knife	5 (1%)	6 (4%)	8 (2%)	15 (4%)	29 (3%)	
	both	4 (1%)	1 (0%)	6 (1%)	7 (2%)	10 (1%)	
	prior papillotomy	283 (62%)	80 (47%)	258 (53%)	156 (37%)	621 (68%)	

Balloon dilatation / bougie dilatation	none	321 (70%)	166 (97%)	426 (87%)	305 (73%)	526 (58%)	< 0,001
	balloon dilatation	65 (14%)	3 (2%)	26 (5%)	10 (2%)	244 (27%)	
	bougie dilatation	59 (13%)	2 (1%)	31 (6%)	92 (22%)	63 (7%)	
	both	13 (3%)	0 (0%)	7 (1%)	12 (3%)	77 (9%)	

Number of stents	0	323	104	228	85	377	< 0,001
	1	75	52	181	173	281	
	2	56	15	76	150	240	
	3	4	0	4	11	8	
	4	0	0	1	0	3	

Stent size	8,5 F	66	13	74	142	130	
	10 F	54	33	145	167	312	
	11,5 F	15	20	40	23	89	
	metal stent	0	1	3	2	1	

Contrast injection in pancreatic duct	no	414	144	390	372	824	< 0,001
	yes	44	27	100	47	86	

Pancreas stent	no	453	166	451	412	900	< 0,001
	yes	5	5	39	7	10	

Complication	bleeding	1	1	1	4	15	< 0,001
	swollen papilla	10	13	5	3	17	
	cast	−	−	48	−	69	
	incomplete examination	14	10	11	21	36	
	hypotension < 70 mmHg	12	5	11	8	17	
	bradycardia < 50 bpm	3	1	2	2	7	
	decrease in oxygen saturation < 85%	15	9	17	10	25	

**Table 3:** *Multivariable linear regression analysis to identify independent factors on the propofol dosage*. Dilatation therapy, age, gender, intervention time, body mass index, platelet count, and bilirubin level were independently associated with the propofol consumption. Propofol dose served as the dependent variable. A logarithmic transformation was applied to all metric variables which showed a skewed distribution on the original scale. In case of more than 2 values for categorical variables a dummy transformation was performed. In a first step all factors were tested in univariate models. Subsequently, significant (p < 0.05) variables were entered into a multivariable model and a backward selection based on the Wald statistic was performed. Table 3 depicts the unstandardized coefficients with standard error, p-values, and 95% confidence interval.

	Unstandardized Coefficients		p-value	95,0% Confidence Interval for B	
	B	Std. Error		Lower Bound	Upper Bound
(Constant)	169,980	57,570	,003	56,820	283,140
Dilatation therapy	49,102	19,731	,013	10,318	87,885
Age	-4,195	,561	,000	-5,297	-3,092
Intervention time	6,385	,342	,000	5,713	7,058
BMI	3,483	1,770	,050	,005	6,962
Platelets	,142	,060	,017	,025	,260
Bilirubin	-,055	,026	,036	-,107	-,004
Gender	46,160	17,440	,008	11,880	80,441

## Data Availability

Data were extracted from the clinical and endoscopy data base. Rare data can be supplied. The data used to support the findings of this study are available from the corresponding author upon request.
